# Comparing the Effectiveness of Honey Consumption With Anti-Cough Medication in Pediatric Patients: A Systematic Review

**DOI:** 10.7759/cureus.29346

**Published:** 2022-09-20

**Authors:** Ghadi D Mashat, Mohammad Hazique, Kokab Irfan Khan, Prasana Ramesh, Suthasenthuran Kanagalingam, Zargham Ul Haq, Nishok Victory Srinivasan, Aujala Irfan Khan, Safeera Khan

**Affiliations:** 1 Pediatrics, California Institute of Behavioral Neurosciences & Psychology, Fairfield, USA; 2 Internal Medicine, California Institute of Behavioral Neurosciences & Psychology, Fairfield, USA; 3 Research, California Institute of Behavioral Neurosciences & Psychology, Fairfield, USA; 4 Internal Medicine/Family Medicine, California Institute of Behavioral Neurosciences & Psychology, Fairfield, USA; 5 Medicine and Surgery, California Institute of Behavioral Neurosciences & Psychology, Farifield, USA; 6 General Surgery, California Institute of Behavioral Neurosciences & Psychology, Fairfield, USA

**Keywords:** benzonatate., guaifenesin, antitussives, honey, cough

## Abstract

Coughing is one of the most prevalent symptoms in children presenting at outpatient departments. This systematic review aimed to review previous literature in order to compare the use of honey and antitussive medications for treating coughs in children. Literature was screened across five databases using Medical Subject Heading (MeSH) strategy, keywords, and inclusion and exclusion criteria. The remaining literature was evaluated using a quality appraisal tool checklist. This review includes systematic reviews, ‎‎meta-analyses, randomized controlled trials (RCTs), observational studies, cross-sectional studies, and articles ‎without ‎a defined ‎methodology section. This review suggests that honey is effective in treating children above 12 months of age, while cold and cough medications (CCMs) are safe if administered at therapeutic doses. Since fatalities can occur in children under two years of age, further RCT studies on CCMs are required to establish safety across all age groups.

## Introduction and background

Acute cough occurs more prevalently in children and is mainly caused by upper respiratory tract infections (URTIs) [1]. Children have a common cold on at least six to eight occasions annually [2,3]. Within most URTI cases, the disease remits within 23 days, though several cases do require approximately eight days of medical treatment [4]. Acute cough can affect sleep and quality of life for parents and children [5,6].

A mild viral respiratory infection in children is most often treated at home without using any medicines but Over-the-counter (OTC) drugs can be used to relieve fever and pain if necessary [1]. The Food and Drug Administration (FDA) recommended in 2008 that children under two years old should not use over-the-counter (OTC) medication that contains a decongestant or antihistamine due to concerns regarding efficacy and safety. In addition, the use of cold and cough medications (CCMs) in children below six years is discouraged by the American Academy of Pediatrics [7]. In 2018, the United States (US) FDA did not recommend using CCMs that contain codeine or hydrocodone due to risks of fatal respiratory depression and addiction in children from 0 to 17 years of age [8-11]. In 2019, The National Poison Data System (NPDS) reported a 16.3% increase in child and adolescent fatalities [12].

Honey is safe for treating the common cold in children one year and older [13]. Honey affects the treatment of cough in children and has an antimicrobial effect [13]. Honey also has antiviral, antioxidant, and synergistic effects with antibiotics [14]. KalobaTUSS®, a pediatric cough syrup based on acacia honey, helps reduce the severity and duration of coughs in children [14].

The efficacy of honey in treating pediatric cough, compared to anti-cough medication, remains scarce. However, the study conducted in 2017 by Ayazi and colleagues focused on the comparative analysis of the effectiveness of two types of Iranian honey formulations and diphenhydramine (DPH) on nocturnal coughing and sleep quality in pediatric patients [15]. Following a two-night investigation of a total of 87 pediatric patients suffering from nocturnal coughs split among three study groups: Honey Type 1 (n=42); Honey Type 2 (n=25); and DPH (n=20), all formulations were effective in reducing nocturnal coughs [15]. However, Honey Type 1 was found to have increased effectiveness in comparison to DPH across all nocturnal cough characteristics, with the exception of coughing frequency, while Honey Type 2 was also more effective for all nocturnal cough characteristics in comparison to DPH with the exception of sleep quality improvements [15]. Within a separate randomized-controlled trial (RCT) study conducted by Cohen and colleagues in 2012, three honey formulations (eucalyptus-based/citrus-based /labiatae-based honey) were investigated in comparison to silan date extract control formulation for nocturnal coughs and sleep quality improvements in 300 pediatric URTI cases of age 1-5 years old [16]. The results of this particular study demonstrated that even though all formulations proved to ameliorate symptoms following administration, all honey formulations had a markedly enhanced effectiveness in reducing nocturnal coughing and increasing sleep quality compared to the silan date extract control formulation [16].

This systematic review aims to determine the effect of honey intake and anti-cough medication on cough pediatric patients, in particular regarding effectiveness‎.

## Review

Methods

Search Source and Search Strategy

This systematic review employed the Preferred Reporting Items for Systematic Reviews and Meta-Analyses (PRISMA) 2020 guidelines [17]. The PubMed, Medline, PubMed Central (PMC), Google Scholar, and Cochrane Library databases were searched for data in the recent five years, up to 05/15/2022. Medical Subject Heading (MeSH) strategy was employed, with Keywords used being ‘cough’, ‘honey’, ‘antitussives’, ‘guaifenesin’, and ‘benzonatate’. The study combined the first three keywords with Boolean "AND" and the last three with Boolean "OR ". The MeSH strategy Keywords results being: ("Cough/diet therapy"[MeSH] OR "Cough/drug effects"[MeSH] OR "Cough/drug therapy"[MeSH] OR "Cough/prevention and control"[MeSH] OR "Cough/therapy"[MeSH]) AND ("Honey/therapeutic use"[MeSH] OR "Honey/therapy"[MeSH]) AND "Antitussive Agents/therapeutic use"[MeSH]) OR "Guaifenesin/therapeutic use"[MeSH]) OR "benzonatate" (Supplementary Concept). The selected studies were published in English and included children and adolescents from birth to 18 years.

The databases were also searched using nine keywords: ‘cough’, ‘cold’, ‘clear throat’, ‘honey’, ‘anti-cough medication’, ‘antitussives’, ‘guaifenesin’, ‘menthol cough drops’, and ‘benzonatate’. The first three keywords were combined with Boolean "OR," and the last five were combined with Boolean "OR." Consequently, the first three keywords, ‘honey’, and the last five keywords were combined with Boolean "AND." The result being: ‘Cough OR cold OR clear throat AND Honey AND Anti-cough medication OR antitussives OR guaifenesin OR menthol cough drops OR benzonatate’. Finally, the search included criteria such as the MeSH strategy.

Screening, Inclusion, and Exclusion Criteria

We screened the literature and removed duplicates. The remaining literature was screened by title and abstract. Consequently, we screened the literature in full text. Finally, we used quality assessment tools for relevant literature, which satisfied > 60%. In this systematic review, inclusion criteria included: relevance to the question, publishing date within the last five years, English publications, and articles focusing on the pediatric population (birth to 18-year old). The exclusion criteria comprised unpublished, grey literature, articles studying animals or adult subjects.

Risk Bias Assessment

**Figure 1:**
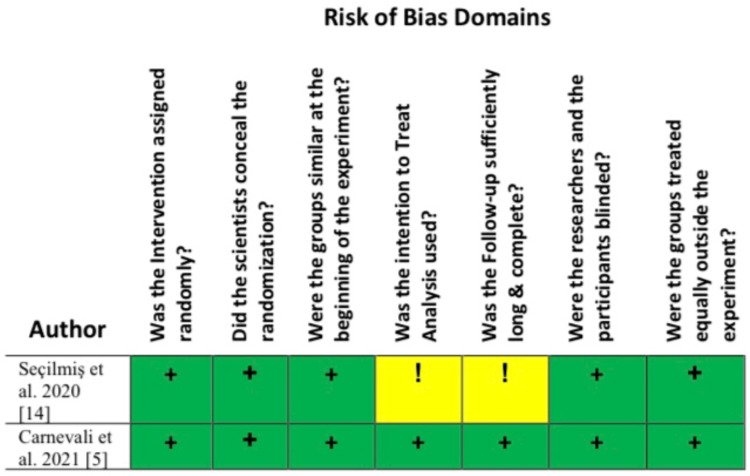
Seven Questions for screening RCT quality‎ RCT: Randomized Controlled trial; Low Risk (+); Some Concerns (!)

Screening for literature bias was performed using quality appraisal tools for 11 studies, as shown in Table 1, while two randomized controlled trial (RCT) studies that employed seven questions to check literature quality (Figure 1) were as follows: (1) Was the intervention assigned randomly? (2) Did the scientists conceal the randomization? (3) Were the groups similar at the beginning of the experiment? (4) Was the intention to Treat Analysis used? (5) Was the follow-up sufficiently long and complete? (6) Were the researchers and the participants blinded? (7) Did the treated groups have similar opportunities to get the intervention, follow-up intensity, and quality of care? Overall, 13 studies with a > 60 % satisfactory quality rate were included in this systematic review.

**Table 1:** Quality Assessment Tools ‎ AMSTAR:  Assessment of Multiple Systematic Reviews; SANRA: Scale for the Assessment of Narrative Review Articles; RCTs: Randomized controlled Trials

Study Types	Number	Quality Assessment Tools
Systematic reviews and meta-analyses	2	AMSTAR checklist
Observational studies	3	Newcastle-Ottawa tool
Cross-sectional study	1	Axis scale
RCTs	2	7-question bespoke screening protocol
Research articles without a clear method section	5	SANRA checklist

Results

Study Search Outcome

**Figure 2:**
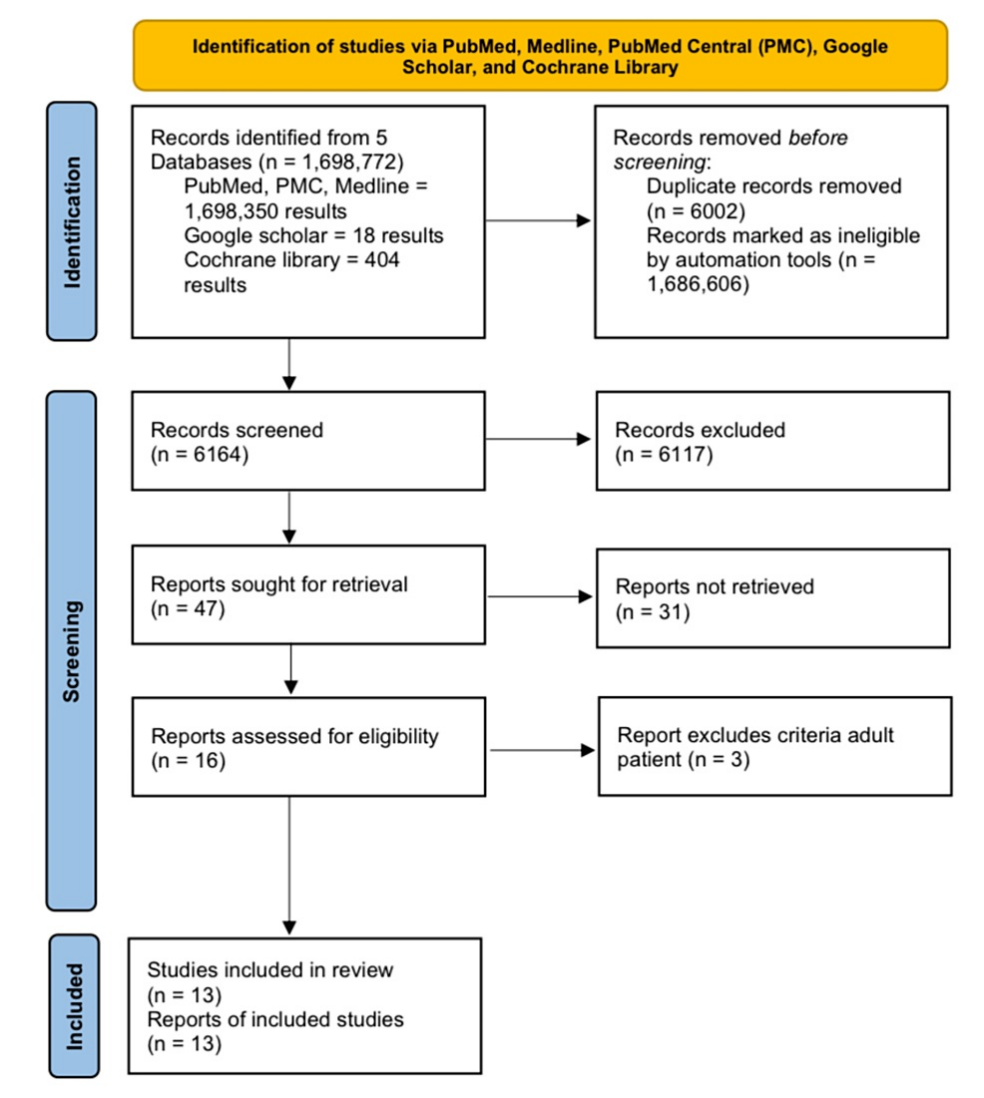
PRISMA Flow Diagram depicting the screening process for This Review‎ PRISMA: Preferred Reporting Items for Systematic Reviews and Meta-Analyses; PMC: PubMed Central

Using the keywords and the MeSH, this study found 1,698,772 studies across all searched databases (PubMed, Medline, ‎PMC, Google Scholar, and Cochrane Library).‎ The number of ‎results returned was ‎6164, after removing ‎6002‎ as duplicated studies and ‎1,686,606‎ as ineligible. Consequently, this study screened the titles and abstracts of 47 studies and found that 31 studies were of relevance. Consequently, 16 studies were identified by screening full-text versions. Consequently, three adult studies out of 16 studies were excluded. Finally, after checking the quality appraisal, this study included 13 studies in this systematic review that satisfied > 60% of the quality and eligibility criteria. This review found four articles on honey, including two systematic reviews and meta-analyses/ RCTs. The remaining were related to cold and cough medications (CCMs) and fatalities, including three observational studies, one cross-sectional study, and five articles without ‎a ‎clear method section.‎ This PRISMA-based screening process is shown below in Figure 2.

Study Characteristics

This review collected limited data, especially for CCMs in children, including one cross-sectional study and three research articles without ‎a clear methods section‎. There were no RCTs /systematic reviews related to CCMs. The results of 13 literature articles included in this systematic review are shown in Table 2.

**Table 2:** The Effect of Honey, CCMS, and Fatalities in Treating Pediatric Coughs ‎ RCT: Randomized Controlled Trial; URTI: Upper Respiratory Tract Infections; CMs: Cough and Cold Medications

Author and Year of Publication	purpose	Drug/ Intervention	Number of patient/Studies	Type of Study	Result/ Conclusion
Abuelgasim et al. 2021 [13]	To evaluate the efficacy of honey in relieving URTI	Honey	14 studies In adult and pediatrics (eight out of 14 studies were in pediatrics)	Systematic review and meta-analysis	Honey was effective in relieving URTI symptoms
Oduwole et al. 2018 [18]	To review the impact of honey on treating children with acute cough in ambulatory settings	Honey	Six studies In 12 months to 18 years	Systematic review and meta-analysis	Honey reduced the cough
Seçilmiş et al. 2020 [14]	To find improvement in treating of bacterial and viral infections in children with URTI	Mixture, placebo, antibiotic + mixture, antibiotic alone	104 children in five‒12 years	RCT	The bee products were effective in treating symptomatic URTI
Carnevali et al. 2021 [5]	To determine Effectiveness and safety of KalobaTUSS® in cough treatment	KalobaTUSS® which contain acacia honey	106 children in three to six years	RCT	KalobaTUSS® decreased cough in children
Halmo et al. 2021 [19]	To report fatalities among children who exposed to CCMs	CCMs	180 eligible fatalities in Children <12 years	Observational Study	40 out of 180 were considered related to CCMs
Green et al. 2017 [20]	To represent the CCMs safety profile in children	CCMs	3251 cases in children <12 years	Observational Study	Adverse events following the CCMs were minute in children
Jurca et al. 2017 [21]	To find cough prevalence among children	questionnaires	7670 children in 12 months to 18 years	Observational Study	69% of coughs are related to cold. Cough is most common in males during the first ten years
Chua et al. 2021 [22]	To evaluate Safety Communications, relate to a prescription change of ‎Codeine or ‎Hydrocodone ‎ CCMs in ‎Children and ‎Adolescents	Codeine or Hydrocodone CCMs	1145357 prescriptions in children 0 -17 years	Cross-Sectional Study	The Codeine and Hydrocodone ‎CCMs prescription ‎decreased between ‎‎2014 to 2019‎
Burns et al. 2021 [12]	To assess fatality behind the CCMs in children	surveillance system	Fatality in children <12 years in 2008-2016 and most in children <2 years for a non-medical purpose [18].	Research Article Without Clear Method Section	CCMs were related to > 40% of fatalities [18].
Van Driel et al. 2018 [23]	To seek information related to effective treatment of common cold to children and adults	-	-	Research Article Without Clear Method Section	The evidence of using saline drops was low in the Cochrane review and could be safe in young children [22].
DeGeorge et al. 2019 [4]	To guide the common cold treatment in children and adults	-	-	Research Article Without Clear Method Section	The safety of honey to treat children one year and older for common cold
Palmu et al. 2022 [6]	To show a significant decrease in the prescription of cough medicine in children	Followed Prescription of cough medicine in the electronic health record from 2014 to 2020 [23-24].	In children 0–15 years [23-24].	Research Article Without Clear Method Section	There was a significant decline in the prescription of cough medicine in children [24].
Korppi 2021 [1]	To recommend not using CCMs in children	-	-	Research Article Without Clear Method Section	Honey effectively treated cough in children >12 months [17].

Discussion

This systematic review studied the effectiveness of honey in comparison to CCMs for treating children with cough. ‎Additional knowledge on the actual clinical effectiveness of honey formulations would be essential for ‎aiding in shifting global prescribing trends away from CCMs and also eventually decreasing mortality ‎rate among pediatric mortalities due to CCM use.‎‎

The Effects of Using CCMs to Treat Children With Cough

The cough prevalence in children is a function of their sex and age. In the first decade of life, coughs were found to be more prevalent in boys than in girls, though such variations decreased once children reached the early teenage phase and were reversed by the age of 14 years [21]. Observational studies from Leicestershire, United Kingdom (UK), across 7670 children aged one to 18 years, suggested a cough prevalence of 69% associated with a cold, 34-55% not associated with a cold, and 25% with nocturnal coughing [21]. These studies indicated that cough prevalence was high in children, exacerbated by exercise and allergens including dust and pollen, compared to children who had nocturnal coughing or cough associated with a cold. Cough is more prevalent in males during the first 10 years than in females, though less within early teens and reversed after 14 years old [21]. The Academy of Pediatrics recommended that children under six years should not use CCMs [7]. Another study showed that children under four years had not used CCMs [4]. There were not enough trials in children under 12 years with a prevalent cold. However, the studies showed safe administration of nasal saline drops, honey (in children above one year), acetylcysteine, intranasal ipratropium, and ointment containing eucalyptus oils and camphor [4,24-27]. Several studies determined the adverse effects of antihistamines, decongestants, antitussives, an antitussive plus bronchodilator, intranasal corticosteroids, and oral prednisolone in children [4,24-27]. In addition, fewer trials were related to risk and reduced effectiveness of decongestant medication in children < 12 years of age [23].

A Cochrane review of two RCTs in 94 members compared oral or intranasal ‎‎decongestants with placebo. The review suggested three to four doses over five or 10 ‎‎days will help reduce nasal congestion. In seven ‎‎RCTs, however, among 11195 participants, no difference was found between decongestants and placebo (these studies did not compare the route ‎‎of administration) [25]. Another Cochrane review of four RCTs among 1466 participants detected ‎‎that antihistamines would relieve rhinorrhea and sneezing compared to placebo. In two RCTs among 375 members, antihistamines did not relieve nasal congestion. Non-‎sedating antihistamines in eight different RCTs showed unaffected relief of ‎congestion (53 members), ‎rhinorrhea (838 members), and sneezing (456 members). In addition, there was no risk when compared to a placebo [26]. There was no evidence in pediatric treatment to use home remedies, and standard treatment included herbs, vitamins, steam, and antibiotics for common colds caused by viral infections [23].

A cross-sectional study (from 2014 to 2019) detected 1,145,357 prescriptions in children and adolescents aged 0 to 17 years. There was a higher percentage of adolescents aged 12-17 (59.4%) using codeine and hydrocodone CCMs [6,28,29]. These were most frequently prescribed by family medicine physicians, pediatricians, and nurse practitioners. Codeine-based CCM ‎prescriptions decreased ‎‎by 90.1%, and hydrocodone ‎CCM prescriptions ‎‎decreased by 75.7% in children 0-17 years old from ‎‎2014 to 2019 [22].‎ Prescription of CCMs for lower tract infections in children aged 0-15 years in two private clinics from 2014 to 2020 was evaluated [6,28,29]. Consequently, during 2017-2020, a follow-up study in children 0-15 years old was carried out with the goal of erasing the prescription of CCMs in children [6]. A significant ‎decrease in CCM prescriptions was noted following an initiative to reiterate prescription guidelines for children 0-15 years old by sending personalized letters or making phone calls [6].

Fatalities Among Children Who Used CCMs

In observation studies, there were 4202 cases; 3251 of these fatal cases were due to a CCM [20]. The highest percentage of exposure was accidental unsupervised, and the most reported product was single ingredient liquid formula [20]. Adverse events such as agitation, hallucinations, and tachycardia were less than twenty percent [20]. There were 20 fatal cases, mostly involving children younger than two years old for which there was no treatment purpose [20]. These studies indicated minimal adverse events and fatalities [20]. Fatalities are not related to treatment purposes of CCMs, though rather to unsupervised children [20]. In comparison, an observational study reported 40 out of 180 fatalities related to children < 12 years old. The most prevalent cause of fatality was diphenhydramine ingestion [19]. Furthermore, CCM-related fatalities, primarily among young children, were given by a caregiver for non-medical purposes. Seven fatal cases were associated with intentional use of sedation [19]. One study reported that from 2008 to 2016, > 40% of CCM-associated fatalities occurred in children < 12 years old [12,19]. The fatalities were rare, though typically occurred in children under two years old and for non-treatment purposes [12,19]. The most prevalent cause of fatalities involved diphenhydramine, an antihistamine [12,19]. ‎In 2019, Poison Control Centers reported an increase in fatality rates by 16.3% in children < 20 days-old [30].

Effectiveness of Honey in Treating Pediatric Coughs

Overall, 13 studies were shortlisted following this investigation on honey's effectiveness in comparison to CCM therapeutic options (Table 2). Upon analysis of these studies, multiple findings could be extrapolated accordingly. Most importantly, two articles recognized the importance and effectiveness of honey formulations in successfully treating upper respiratory tract infections (URTIs). The first was a systematic review and meta-analysis comprising 14 studies, eight of which described that honey relieved symptoms of URTI more effectively than usual care and helped decrease the level of antimicrobial resistance [13]. The second article was an RCT that included children from five to 12 years old. The total number of children was 104 (59 boys, 45 girls), of which 50 patients were assessed for bacterial infections and 54 for viral infections [14]. The sample population was divided into four groups: mixture product compared to the placebo group for viral infections, and antibiotic + mixture compared to an antibiotic alone group for bacterial infections. This showed earlier enhancement in children who had a viral infection and were treated with a combined product compared to another group. Furthermore, the bacterial infection group treated with antibiotic + mixture group showed a significant decrease in CARIFS (Canadian Acute Respiratory Illness and Flu Scale) scores, indicating the relief of symptoms using bee product [14].

Honey was also effective in treating coughs in children as young as just over 12 months of age [4,18]. According to the study by Oduwole and colleagues, 899 children were enrolled in RCTs, and three other studies, which included 331 children [18]. The age included from 12 months to 18 years ‎old. It compared honey with dextromethorphan, diphenhydramine, salbutamol, bromalin, no treatment, and placebo. Using a 7-point Likert scale, it was shown that honey was superior to ‎diphenhydramine, with no treatment or placebo, and honey could be superior to diphenhydramine. However, honey could have the same effect as dextromethorphan. When honey was compared to placebo or salbutamol, honey relieved cough symptoms if it relented within the first three days, though following from this, all would have the same effect. ‎Using a 5-point cough scale, honey and bromalin mixed with honey had equivalent effects, with no difference in treating cough symptoms [18]. In addition, one RCT used KalobaTUSS®, including acacia honey and another plant extract. Among 106 children (50.8% boys, 49.2% girls), at the age of three to six years, 54 children received the KalobaTUSS® syrup, and 52 received a placebo. Children who used KalobaTUSS® revealed a decrease in cough during the day and night, compared with another group, using a 6-points Likert scale. The effectiveness of KalobaTUSS® in cough treatment and duration of cough in children was suggested by Carnevali and colleagues [5].

This systematic review found CCMs safe and effective for medical purposes and cough treatment. It showed that fatalities occurred more frequently in children under 12 months of age who received CCMs for non-medical purposes‎. However, this age group could incur botulism if they receive honey. We must protect them by educating the parents or developing medication that helps this age group.

Limitations

We found 13 studies, two RCTs, related to honey. However, there were no RCTs related to CCMs, perhaps since important literature could have been excluded using our criteria for articles published in English over the last five years. Furthermore, since all fatalities in the literature ‎focused on < 12 years of age, data regarding fatalities among other groups were absent.

## Conclusions

Cough is a prevalent symptom ‎in children, causing major discomfort. This systematic review aimed to detect whether, compared to cold and cough medications‎ (CCMs), honey intake was adequate and safe in children with a cough. We found that honey was effective in treating children above 12 months of age, mainly if used in the first three days of cough symptoms. Honey can lead to botulism if used in children < 12 months. CCMs can be used if children are > six years old, with the exception of antihistamines, decongestants, and antitussives. Moreover, most case fatalities were not related to a therapeutic dose. In essence, such additional knowledge on the actual clinical effectiveness of honey formulations is essential for aiding in shifting the global prescribing trends away from CCMs, and also eventually decrease the mortality rate among pediatric mortalities due to CCM use.
